# Real-world interpretation of procalcitonin to guide antibiotic prescribing: a retrospective cohort study with regression discontinuity analysis

**DOI:** 10.1017/ash.2025.72

**Published:** 2025-05-28

**Authors:** Leela Chockalingam, Thomas Mee, Tiffany Gardner, Eric Grimm, Amiran Baduashvili

**Affiliations:** 1 Division of Hospital Medicine, Northwestern University Feinberg School of Medicine, Chicago, IL, USA; 2 Internal Medicine Residency Program, University of Colorado School of Medicine, Aurora, CO, USA; 3 Pulmonary & Critical Care Fellowship Program, Massachusetts General Hospital/Beth Israel Deaconess Medical Center, Boston, MA, USA; 4 Division of Hospital Medicine, University of Colorado - Anschutz Medical Campus, Aurora, CO, USA

## Abstract

**Objective:**

It is unknown how providers are utilizing procalcitonin in the real world to make antibiotic prescribing decisions, and whether procalcitonin can limit harms related to antibiotic misuse. We examined how the probability of receiving antibiotics changed just below and above the pre-specified procalcitonin cut-points. We sought to understand whether providers interpret procalcitonin as a dichotomous or continuous diagnostic test.

**Design:**

Retrospective cohort study.

**Participants:**

We included adult inpatients who had procalcitonin collected as part of routine care. Patients with procalcitonin collected more than 48 hours after the first antibiotic dose, those discharged from the emergency department, or those on an obstetrics service were excluded.

**Methods:**

We used administrative data from the Health Data Compass database (2018-2019) at the University of Colorado Hospital to examine the correlation of pre-specified procalcitonin cut-points (0.1, 0.25, and 0.5 ng/mL) with the antibiotic treatment decisions using a regression discontinuity analysis (RDA), stratified by level of care. We constructed receiver operating characteristic (ROC) curves depicting the relationship between procalcitonin level and antibiotic prescribing. We performed sensitivity analyses by varying bandwidth for RDA.

**Results:**

The study included 4383 patients. A total of 68.9% received a full antibiotic course. RDA did not demonstrate any discontinuity at the pre-specified cut-points. However, sensitivity analyses showed a potential discontinuity at the 0.25 ng/mL cut-point in the ICU subgroup. The ROC curves were consistent with the RDA findings.

**Conclusions:**

This study suggests that most clinicians in real-world settings interpret procalcitonin as a continuous diagnostic test when prescribing antibiotics.

## Introduction

Among hospitalized patients, an estimated 30 to 50% of antibiotic therapies may be unnecessary.^1^ Several factors lead to antimicrobial overuse, including challenges with diagnosing infection, over-treating colonizing bacteria, lengthy treatment durations, and fear of under-treating.^1–4^ Antibiotic overuse has been linked with drug reactions, Clostridioides difficile infections, and increased healthcare expenditures.^5–7^ Conversely, a systematic review and meta-analysis of 37 studies demonstrated that a delay in appropriate antibiotic use is associated with increased mortality.^8^ Since antibiotics are often initiated empirically based on clinical suspicion and without a clear reference standard for bacterial infection, the role of biomarkers such as procalcitonin have been explored in empiric treatment decisions.

Multiple randomized controlled trials (RCTs) have demonstrated that procalcitonin algorithms may reduce antibiotic use without compromising patient outcomes, although antibiotic use was not consistently lower in all studies.^9–13^ However, even in these controlled settings, clinicians followed the procalcitonin algorithms only around 50%–60% of the time, likely incorporating history, vital signs, symptoms, and other lab values into their decisions.^14,15^ In real-world settings, it is unclear how closely clinicians’ antibiotic decisions align with procalcitonin levels.^16^

The Food and Drug Administration cleared the use of select procalcitonin assays to guide antibiotic initiation and discontinuation in lower respiratory tract infections, as well as discontinuation in sepsis. Early reviews recommended using the following cut-points to guide decisions: 0.1, 0.25, and 0.5 ng/mL.^12^ However, the 2019 American Thoracic Society (ATS) and Infectious Diseases Society of America (IDSA) guidelines for community-acquired pneumonia (CAP) did not endorse the use of procalcitonin cut-points for initiating antibiotics.^17^ Instead, the guidelines suggested there is no clear cut-point that reliably differentiates bacterial and viral causes of CAP, but higher procalcitonin values do correlate with a higher likelihood of bacterial infection.^17,18^ These guidelines imply that the probability of a bacterial infection rises gradually with the procalcitonin level and that procalcitonin interpretation should not be dichotomized to guide antibiotic usage. It has been previously demonstrated that non-dichotomous interpretation of continuous diagnostic tests (ie, B-type natriuretic peptide, D-dimer, and troponin, among many others) contributes to diagnostic excellence.^19–24^

It is unclear whether most providers interpret procalcitonin in a dichotomous or continuous manner. To address this uncertainty, we conducted a single-center retrospective cohort study of adult inpatients, using regression discontinuity analysis (RDA) to investigate whether previously recommended procalcitonin cut-points have a significant impact on antibiotic treatment decisions, implying a dichotomous interpretation.

## Methods

### Study design, setting, and data sources

We conducted this observational study using administrative data collected from the Health Data Compass (Compass) database at the University of Colorado Hospital (UCH) in Aurora, Colorado, United States. Compass is a multi-institutional data warehouse affiliated with the University of Colorado Health System. It contains inpatient and outpatient electronic health records (EHR) including patient- and encounter-level data from two EHR systems, claims data, and the Colorado death registry. Patients’ clinical variables were collected as part of their routine medical care. Storage of identifying patient information was minimized. The Colorado Multiple Institutional Review Board (COMIRB # 20-0135) reviewed and approved this study protocol. Informed consent was waived given the retrospective design. This manuscript follows the STROBE reporting checklist for cohort studies.^25^

### Participants

Eligible participants included all consecutive adults (≥18 yr) admitted for inpatient care at the UCH between January 1, 2018, and December 31, 2019, and had procalcitonin collected as part of routine medical care. Patients with a positive pregnancy test or those admitted to an obstetrics service were excluded. We only included the first inpatient encounter for patients with multiple eligible admissions during the study period. We excluded encounters in which procalcitonin was collected more than 48 hours after the first antibiotic dose, as we presumed that prescribers were intending to use procalcitonin to inform antibiotic de-escalation rather than initiation. We excluded encounters where participants were discharged directly from the Emergency Department (ED), as we could not reliably ascertain antibiotic prescriptions at ED discharge.

### Procalcitonin assay

During the study period, the laboratory services used Abbott Architect i2000SR procalcitonin chemiluminescent microparticle immunoassay. We only considered the first procalcitonin result for encounters where multiple procalcitonin assays were collected. The lab reported results using ng/mL units, dichotomized at 0.1 ng/mL, with the results above 0.1 ng/mL reported as abnormal and highlighted in red. Procalcitonin results included explanatory comments to guide antibiotic prescribing (Supplemental Figure 1a–b).

### Variables

The collected variables included patient age, sex, race, hospital length of stay (LOS), highest level of care (LOC) (floor, step-down unit, or intensive care unit (ICU)), procalcitonin result and time of collection, full antibiotic administration history, presence of an antibiotic prescription at discharge, hospital discharge disposition, International Classification of Diseases (ICD)-9 and ICD-10 billing and encounter codes, and primary discharge diagnosis. Primary discharge diagnoses were categorized into noninfectious diagnoses and types of infections.

### Treatment decisions

We assessed if each patient had received a full course, a partial course, or no antibiotics. A full course was defined as any of the following: (a) receiving antibiotics for at least four calendar days within any five-day period during hospitalization, (b) a prescription of a minimum four-day antibiotic course at discharge, or (c) continuation of the inpatient antibiotic regimen via a discharge prescription, totaling to at least four days. The four-day cutoff for a full course was based on authors’ clinical experience, as three-day antibiotic courses were rarely used for urinary or lower respiratory tract infections at our center. Additional details on treatment decision categorizations are available in Supplement Table 1. We combined the no-treatment and partial-treatment groups, as providers in both scenarios decided a full course of antibiotics was unwarranted and either did not prescribe or stopped antibiotics based on clinical and laboratory values, including procalcitonin.

### Outcomes

We investigated how procalcitonin cut-points influenced clinical decision-making regarding antibiotic prescriptions. We pre-specified the cut-points based on the initial procalcitonin utilization recommendations, namely, 0.1, 0.25, and 0.5 ng/mL.^12^ We assessed the changes in probability of receiving antibiotics around these pre-specified cut-points. Additionally, we stratified the analysis by the LOC.

To explore the impact of procalcitonin cut-points on treatment decisions, we used RDA, which permits potential causal inference by leveraging natural variation around a specific cut-point. Procalcitonin assay interpretation has analytical, intra- and inter-individual variation between 10 and 30%.^26,27^ Thus, for the cut-point of 0.25 ng/mL, we assume the procalcitonin values that cluster just above (ie, 0.25–0.29 ng/mL) and just below the cut-point (ie, 0.20–0.24 ng/mL) reflect random laboratory analytical or intra-personal variation, and are likely unrelated to confounders such as disease severity or specific diagnoses. A discontinuity in the probability of antibiotic prescribing around a cut-point would suggest a causal effect on antibiotic treatment decisions.

### Statistical analysis

We used descriptive statistics to report participant and encounter-level variables. We compared encounters with full antibiotic courses to encounters with partial or no antibiotic course using chi-squared or Fisher’s exact tests for categorical variables and t-tests with unequal variance for continuous variables. We used the pre-specified procalcitonin cut-points as the treatment variable, and the actual procalcitonin level as the running variable. For RDA, we used a bandwidth of 0.05 ng/mL for the cut-points of 0.1 and 0.25 ng/mL, and a wider bandwidth of 0.1 ng/mL for the cut-point of 0.5 ng/mL based on greater analytical test variability at higher values.^26,27^ When stratifying the analysis by the LOC, we used patients within the same LOC with procalcitonin level lower than the cut-point as the reference. Sensitivity analyses were done using different bandwidths of 0.03, 0.05, and 0.10 ng/mL for each cut-point.

Additionally, we plotted a receiver operating characteristic (ROC) curve using procalcitonin as the independent variable and the antibiotic treatment course dichotomized into full versus partial or no antibiotics as the dependent variable. We used this ROC curve to visually estimate the cut-point effect on treatment decisions. We labeled the ROC curve with the pre-specified procalcitonin cut-points and additional cut-points at 1 and 5 ng/mL and visually evaluated for the presence of inflection points, which would suggest a potential cut-point impact on antibiotic treatment decisions. We first plotted an ROC curve for all patients and then stratified it by the LOC. Area under the curve (AUC) was reported for each ROC curve. ROC curves were generated and plotted using the packages pROC (v1.16.2) and ggplot2 (v3.3.5), and RDAs were performed using the packages rddtools (v1.6.0) and emmeans (v1.7.5) in R version 3.6.3.^28–32^

## Results

### Participants

Of the 6336 encounters evaluated, 4383 met the eligibility criteria. The reasons for exclusion are detailed in Supplement Figure 1. The mean participant age was 61.4 years (SD 16.4), 42.5% were female, 16% identified as Black or African American, and 62.7% as white (Table 1). A total of 3,018 (68.9%) patients received a full course of antibiotics. Patients who received full course of antibiotics had longer median LOS (six vs four days), were more likely to receive ICU level care (38.7% vs 20.7%), had higher mortality (9.3% vs 1.6%), and were more likely to be discharged to a rehab facility (20.1% vs 14.1%). Noninfectious primary discharge diagnoses were the most common diagnosis types in both groups, although less common among those who received a full antibiotic course (52.8% vs 85.1%, Table 2). Among patients who received a full course of antibiotics, sepsis was the most common infectious discharge diagnosis (28.2%), followed by lower respiratory tract infections (8.3%).

**Table 1.** Patient/encounter baseline characteristics, stratified by the antibiotic treatment decisions

Patient/encounter characteristic	Full Course of Antibiotics (n = 3,018)	Incomplete Course/No Antibiotics (n = 1,365)	Total (n = 4,383)	*P*-value *
**Patient age (Years)**				
Mean (SD)	61.2 (16.2)	61.7 (17.1)	61.4 (16.4)	0.39
Median (IQR)	62 (51, 73)	63 (52, 74)	63 (51, 73)	
**Patient sex**				
Female	1227 (40.7%)	635 (46.5%)	1862 (42.5%)	0.00031
Male	1791 (59.3%)	730 (53.5%)	2521 (57.5%)	
**Patient Race**				
American Indian and Alaska Native	20 (0.7%)	5 (0.4%)	25 (0.6%)	0.0035
Asian	97 (3.2%)	45 (3.3%)	142 (3.2%)	
Black or African American	439 (14.5%)	261 (19.1%)	700 (16.0%)	
Native Hawaiian and Other Pacific Islander	12 (0.4%)	5 (0.4%)	17 (0.4%)	
White or Caucasian	1939 (64.2%)	810 (59.3%)	2749 (62.7%)	
Multiple/Other/Unspecified	511 (16.9%)	239 (17.5%)	750 (17.1%)	
**Length of stay (days)**				
Mean (SD)	11.8 (19.0)	5.6 (7.6)	9.9 (16.6)	<0.00001
Median (IQR)	6 (3, 14)	4 (2, 7)	5 (3, 11)	
**Highest level of care**				
Intensive care unit	1167 (38.7%)	283 (20.7%)	1450 (33.1%)	<0.00001
Stepdown	469 (15.5%)	230 (16.8%)	699 (15.9%)	
Floor	1344 (44.5%)	826 (60.5%)	2170 (49.5%)	
Missing	38 (1.3%)	26 (1.9%)	64 (1.5%)	
**Hospital discharge disposition**				
Discharge home/self	1963 (65.0%)	1064 (77.9%)	3027 (69.1%)	<0.00001
Rehab/facility-based	607 (20.1%)	192 (14.1%)	799 (18.2%)	
Death	284 (9.4%)	22 (1.6%)	306 (7.0%)	
Hospice	106 (3.5%)	43 (3.2%)	149 (3.4%)	
Patient-directed	25 (0.8%)	29 (2.1%)	54 (1.2%)	
Acute care hospital	30 (1.0%)	13 (1.0%)	43 (1.0%)	
Admitted from ED	3 (0.1%)	2 (0.1%)	5 (0.1%)	

SD, standard deviation; IQR, interquartile range; ED, emergency department.

* *P*-values were calculated using chi-squared or Fisher’s exact tests for categorical variables and unequal variances t-tests for continuous variables.

**Table 2.** Discharge diagnoses, stratified by antibiotic treatment decisions

Primary Discharge Diagnosis Category	Full Course of Antibiotics (n = 3,018)	Incomplete Course/No Antibiotics (n = 1,365)	Total (n = 4,383)
Noninfectious	1595 (52.8%)	1162 (85.1%)	2757 (62.9%)
Sepsis	852 (28.2%)	74 (5.4%)	926 (21.1%)
Lower respiratory infection	249 (8.3%)	23 (1.7%)	272 (6.2%)
Viral Diseases	96 (3.2%)	85 (6.2%)	181 (4.1%)
Genitourinary infection	39 (1.3%)	0 (0.0%)	39 (0.9%)
Intra-abdominal infection	33 (1.1%)	3 (0.2%)	36 (0.8%)
Unspecified infection	29 (1.0%)	2 (0.1%)	31 (0.7%)
Abscess	29 (1.0%)	0 (0.0%)	29 (0.7%)
Bloodstream infection	24 (0.8%)	1 (0.1%)	25 (0.6%)
Brain/spine infection	19 (0.6%)	1 (0.1%)	20 (0.5%)
Cardiac infection	17 (0.6%)	1 (0.1%)	18 (0.4%)
Skin infection	15 (0.5%)	1 (0.1%)	16 (0.4%)
Upper Respiratory	6 (0.2%)	7 (0.5%)	13 (0.3%)
Bone/joint infection	12 (0.4%)	0 (0.0%)	12 (0.3%)
Fungal infection/parasites	1 (0.0%)	5 (0.4%)	6 (0.1%)
Other	2 (0.1%)	0 (0.0%)	2 (0.0%)

### Procalcitonin levels

Patients who received a full antibiotic course had a higher median procalcitonin level (0.29 ng/mL, IQR 0.1–1.24) compared to those who did not (0.08 ng/mL, IQR 0.04–0.19), and a higher incidence of initial procalcitonin level over 0.5 ng/mL (39.1% vs 10.2%). Additional details about procalcitonin levels are provided in the Supplement Table 2.

### Regression discontinuity analysis

The number of patients with procalcitonin levels clustered around 0.1, 0.25, and 0.5 ng/mL was 1161, 383, and 275, respectively. Participant and encounter characteristics for those patients are detailed in Table 3. There were no statistically significant differences in the baseline variables among those just below and above the specified cut-points with only one exception. The LOS was shorter among patients with procalcitonin in the 0.51 to 0.6 ng/mL range, compared to those with procalcitonin between 0.41 and 0.5 ng/mL (8.55 vs 12.49 d, *P* = 0.013). RDA did not demonstrate any statistically significant discontinuity when including all patients or when stratifying by the LOC at any of the pre-specified thresholds (Table 4). The sensitivity analyses with varying bandwidths did not show discontinuity, except a discontinuity around the 0.25 cut-point (bandwidths +/− 0.03 or 0.1 ng/mL) in the ICU subgroup, and around the 0.25 cut-point (bandwidth +/− 0.1 ng/mL) in all LOC. (Supplement Table 3).

**Table 3.** Encounter characteristics, stratified by the categorization for the regression discontinuity analysis

	Procalcitonin cut-point = 0.1 ng/mL (Bandwidth +/− 0.05)			Procalcitonin cut-point = 0.25 ng/mL (Bandwidth +/− 0.05)			Procalcitonin cut-point = 0.5 ng/mL (Bandwidth +/− 0.1)		
	0.06–0.10; n = 720	0.11–0.15; n = 441	Total (n = 1161)	0.2–0.24; n = 238	0.25–0.29; n = 145	Total (n = 383)	0.41–0.50; n = 155	0.51–0.60; n = 120	Total (n = 275)
**Patient Age (Years)**									
Mean (SD)	62.9 (16.4)	62.6 (15.8)	62.7 (16.2)	60.7 (16)	60.7 (15.3)	60.7 (15.7)	61.1 (16.2)	60.8 (15.6)	61 (15.9)
Median (IQR)	64 (53,75)	64 (53,73)	64 (53,74)	62 (51,72)	63 (51,71)	62 (51,72)	61 (49.5,73)	64 (52.8,71)	63 (51,72)
**Patient Sex n (%)**									
Female	331 (46.0)	186 (42.2)	517 (44.5)	97 (40.8)	58 (40.0)	155 (40.5)	57 (36.8)	49 (40.8)	106 (38.5)
Male	389 (54.0)	255 (57.8)	644 (55.5)	141 (59.2)	87 (60.0)	228 (59.5)	98 (63.2)	71 (59.2)	169 (61.5)
Length of stay (days)*^a^									
Mean (SD)	8.23 (14.86)	8.65 (11.02)	8.39 (13.53)	9.95 (10.96)	9.7 (10.79)	9.85 (10.88)	12.49 (16.97)	8.55 (8.5)	10.77 (14.04)
Median (IQR)	4.5 (3, 8)	5 (3, 10)	5 (3, 9)	6 (3,13.8)	6 (3,11)	6 (3,12)	7 (4,14)	6 (3,10)	6 (4, 11.5)
**Highest level of care, n (%)**									
ICU	173 (24.0)	114 (25.9)	287 (24.7)	77 (32.4)	52 (35.9)	129 (33.7)	57 (36.8)	36 (30.0)	93 (33.8)
Stepdown	124 (17.2)	75 (17.0)	199 (17.1)	33 (13.9)	28 (19.3)	61 (15.9)	25 (16.1)	19 (15.8)	44 (16.0)
Floor	410 (56.9)	241 (54.6)	651 (56.1)	125 (52.5)	62 (42.8)	187 (48.8)	69 (44.5)	64 (53.3)	133 (48.4)
Unspecified	13 (1.8)	11 (2.5)	24 (2.1)	3 (1.3)	3 (2.1)	6 (1.6)	4 (2.6)	1 (0.8)	5 (1.8)

SD = standard deviation, IQR = interquartile range, ICU = intensive care unit.

* *P*-values were calculated using chi-squared or Fisher’s exact tests for categorical variables and unequal variances t-tests for continuous variables. All *P*-values were insignificant except for the one reported below.

a *P*-value for 0.5 cut-point significant at 0.01267.

**Table 4.** Regression discontinuity analyses

Level of care	Cut-point ± bandwidth; comparison; (n)	Odds ratio* (95% confidence Intervals)	*P*-value
All patients	0.1 ± 0.05 ng/mL; 0.06–0.10 vs 0.11–0.15; (n = 1161)	0.69 (0.42 – 1.13)	0.15
	0.25 ± 0.05 ng/mL; 0.20–0.24 vs 0.25–0.29 (n = 383)	2.11 (0.83 – 5.40)	0.12
	0.50 ± 0.1 ng/mL; 0.41–0.50 vs 0.51–0.6 (n = 275)	3.57 (0.80 – 16.04)	0.1
Intensive care unit (N = 509)	0.1 ± 0.05 ng/mL; 0.06–0.10 vs 0.11–0.15; (n = 287)	0.63 (0.32 – 1.21)	0.17
	0.25 ± 0.05 ng/mL; 0.20–0.24 vs 0.25–0.29 (n = 129)	2.68 (0.75 – 9.50)	0.13
	0.50 ± 0.1 ng/mL; 0.41-0.50 vs 0.51–0.6 (n = 93)	11.57 (0.92 – 144.81)	0.06
Stepdown (N = 304)	0.1 ± 0.05 ng/mL; 0.06–0.10 vs 0.11-0.15; (n=199)	0.73 (0.36 – 1.50)	0.39
	0.25 ± 0.05 ng/mL; 0.20–0.24 vs 0.25–0.29 (n = 61)	1.47 (0.35 – 6.20)	0.60
	0.50 ± 0.1 ng/mL; 0.41–0.50 vs 0.51–0.6 (n = 44)	5.58 (0.65 – 47.50)	0.12
Floor (N = 971)	0.1 ± 0.05 ng/mL; 0.06–0.10 vs 0.11–0.15; (n= 651)	0.70 (0.40 – 1.20)	0.20
	0.25 ± 0.05 ng/mL; 0.20–0.24 vs 0.25–0.29 (n = 187)	2.05 (0.73 – 5.77)	0.17
	0.50 ± 0.1 ng/mL; 0.41–0.50 vs 0.51–0.6 (n = 133)	2.80 (0.56 – 13.98)	0.21

*Odds ratio provides the ratio of odds of receiving full antibiotic course among patients above versus below the cut-point, within the bandwidth boundaries.

### ROC curve analysis

**Figure 1.**
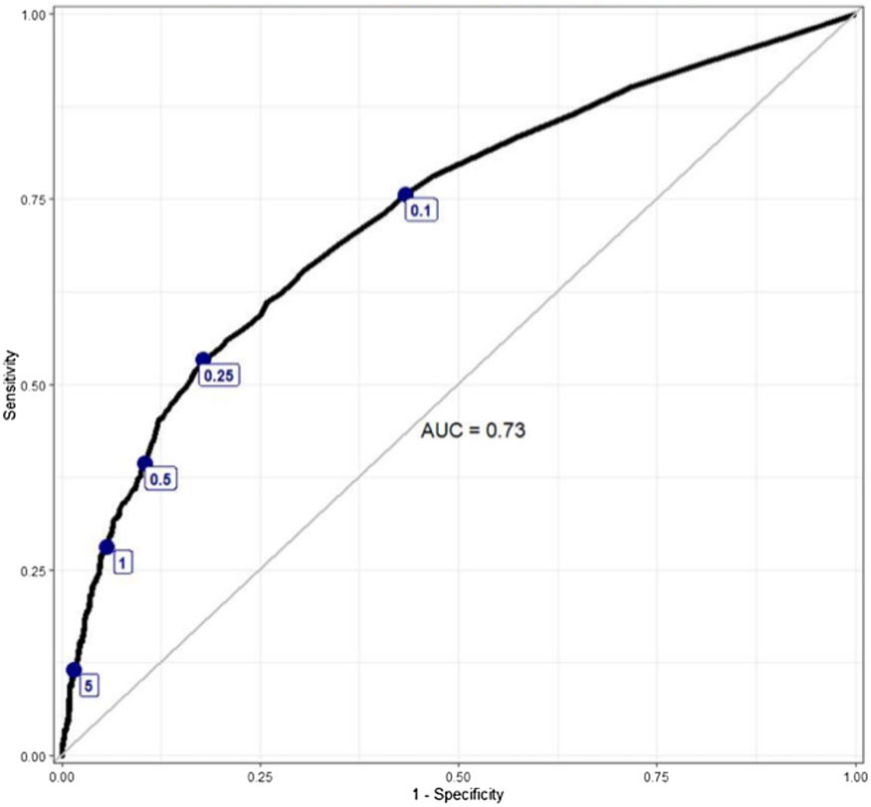
An ROC curve depicting the relationship between antibiotic treatment decisions and procalcitonin level, labeled with specific procalcitonin cut-points.

**Figure 2.**
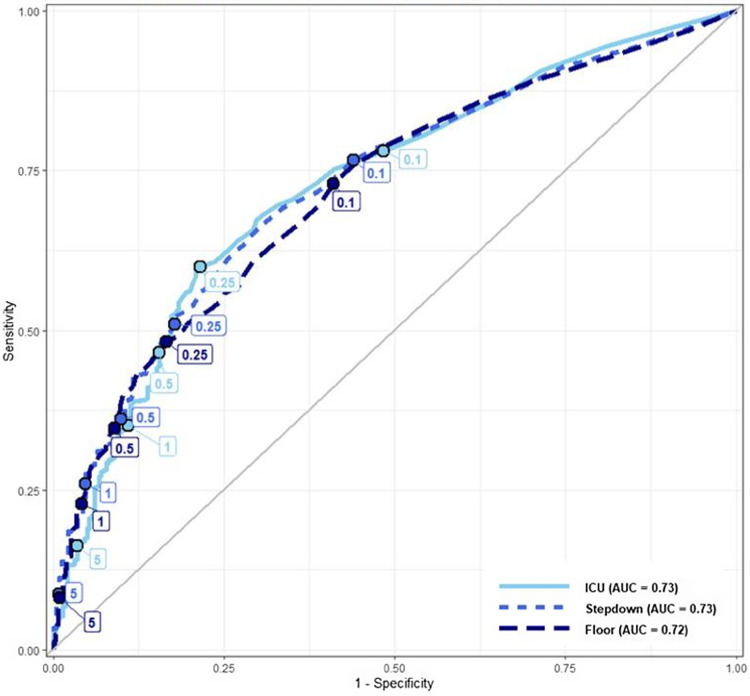
ROC curves depicting the relationship between antibiotic treatment decisions and procalcitonin level, stratified by the highest encounter level of care, each ROC labeled with specific procalcitonin cut-points.

The ROC curves depict the relationship between procalcitonin cut-points and antibiotic treatment decisions for all study patients (Figure 1) and for patients stratified by the LOC (Figure 2). The combined and stratified ROC curves had AUCs between 0.72 and 0.73 (Figures 1–2). The pre-specified (0.1, 0.25, and 0.5 ng/mL) and additional (1.0 and 5.0 ng/mL) cut-points were labeled on each ROC curve. The ROC curves for all patients and those stratified by LOC did not demonstrate any visibly significant slope inflection at the pre-specified cut-points except for the 0.25 ng/mL cut-point in the ICU subgroup (Figures 1–2).

## Discussion

In our study, we observed that the probability of prescribing a full antibiotic course gradually increased with the increasing procalcitonin level. RDA did not reveal any statistically significant discontinuities at any of the pre-specified procalcitonin cut-points and bandwidths, indicating that most providers likely interpreted procalcitonin in a continuous rather than dichotomous manner. One notable exception is a discontinuity in the sensitivity analysis around the 0.25 ng/mL cut-point (bandwidths 0.03 or 0.1 ng/mL) in the ICU patients and around 0.25 ng/mL cut-point (bandwidths 0.1 ng/mL) among all LOC. We did not visually observe significant inflection points at the pre-specified procalcitonin cut-points in the ROC curves, except for the ICU patients around 0.25 ng/mL, consistent with the RDA findings.

We propose several potential explanations for our findings. First, the providers at this institution may be skilled at appropriate use of procalcitonin, relying primarily on the pre-test probability of infection and severity of illness, and only using procalcitonin as an adjunct to their decision-making. Second, the EHR comment accompanying procalcitonin results appropriately cautioned against replacement of clinical judgment with a single procalcitonin value, which may have counteracted the dichotomous interpretation. Third, the providers may have ignored or placed very little weight on procalcitonin value, anchoring their decisions primarily on relevant factors such as history, physical exam, and other laboratory and imaging findings. Fourth, there may be large inter-provider variability in procalcitonin interpretation as was also seen in a study of National Health Service providers, with a minority placing a large emphasis on the specific cut-points, and most others ignoring it, leading to the net neutral effect.^33^ Moreover, the discontinuity in RDA may have been observed if we only looked at specific types of suspected infections, such as lower respiratory tract infections, but our design and sample size did not permit provider-specific or disease-specific evaluation of procalcitonin use.

Regardless of the underlying drivers for the study results, the overall findings provide evidence that most providers at our institution did not use specific thresholds to dictate antibiotic prescribing decisions. Such an approach is consistent with the ATS/IDSA guidelines that acknowledge the absence of a definitive procalcitonin cut-point for distinguishing bacterial from viral infections, yet they recognize a correlation between higher procalcitonin levels and bacterial infections.^17^ The probability of prescribing a full antibiotic course did increase with procalcitonin level. Our ROC curve analyzing antibiotic treatment decisions appears similar in shape and AUC to previously published ROC curves that explore the role of procalcitonin in the diagnosis of bacterial infections.^18,34,35^

Sensitivity analysis did show a potential discontinuity around 0.25 ng/mL in the ICU patients with bandwidths 0.03 and 0.1 and for all patients around 0.25 ng/mL with the 0.1 bandwidth. Higher bandwidths (such as 0.1 for the 0.25 ng/mL cut-point) that are beyond the expected assay’s analytical variability increase the probability of underlying confounding differences between the compared groups, which may lead to the discontinuity. Furthermore, a false positive finding due to multiple hypothesis testing without a *P*-value threshold adjustment is another potential explanation. However, it is conceivable that providers are more likely to rely on a dichotomous cut-point for ICU patients with higher illness severity.

Our study builds on the prior research by Choi et al. revealing that providers often override procalcitonin algorithms when prescribing antibiotics for hospitalized patients.^16^ Prescribers often over-ruled prescribing algorithms in the original procalcitonin RCTs as well.^14,15^ In a recent trial, providers successfully reduced antibiotic prescribing for COVID-19 patients using a procalcitonin algorithm without adversely impacting patient outcomes; notably, they could make discretionary decisions,^36^ implying the procalcitonin value was integrated into the clinician’s overall assessment of the patient’s pre-test probability of having a bacterial infection and patients’ severity of illness. The previously proposed procalcitonin algorithms with one or multiple cut-points are unable to fully accommodate nuanced assessments of infection probability and illness severity. Thus, the concepts of procalcitonin algorithm and algorithm compliance may be unnecessary oversimplifications of complex decision-making and may be of questionable value.

Our study leveraged two unique methodological approaches to examining clinical decision-making: visual examination of ROC curves and RDA. Unlike conventional ROC curves that use presence or absence of a diagnosis as the dependent variable, we used a clinical decision (antibiotic prescribing) as the dependent variable and evaluated the role of the independent variable (procalcitonin) in predicting the clinical decision. Such an approach can be extended to evaluating how other laboratory assays impact clinical decision-making.

The use of the RDA approach in healthcare and epidemiology research is increasing.^37^ RDA helps establish causality with observational data by approximating quasi-randomization and reducing confounding.^38^ It achieves this while analyzing real-world data, which mitigates biases associated with RCTs such as the Hawthorne effect.^39^ While this study design has previously been employed to investigate topics such as the relationship between intravenous contrast and acute kidney injuries, as well as the benefits of early antiretroviral therapy and statins, it remains underutilized in observational clinical research.^38,40^ We used RDA to assess decision-making and explored whether healthcare providers interpret test results dichotomously or continuously, a method that could answer other related clinical questions.

Our study has several limitations. First, this is a single-center study. Clinical decision-making could be linked to local educational, cultural, and operational practices, limiting the study’s generalizability. Second, although we included over 4,000 patients, individual RDA sub-groups had limited sample size, and a larger sample may have revealed discontinuity. Some odds ratios (Table 3) appear large; however, odds ratios can overestimate effect size when the outcome is highly prevalent (68.9% received full antibiotic course).^41^ Third, the RDA assumptions may not have been accurate, leading to residual confounding, although confounding would likely bias the results away from null. Fourth, the use of multiple cut-points may increase the probability of false positive findings, but we did not observe a positive result, i.e., discontinuity at any pre-specified thresholds except in our sensitivity analysis.

Our study demonstrates that higher procalcitonin values are associated with a greater likelihood of receiving a full antibiotic course. However, it suggests that providers are not overly reliant on procalcitonin cut-points to inform antibiotic prescribing and recognize the need for a holistic assessment of various clinical factors. Future RDA-based studies would benefit from a larger sample size and multi-institutional collaboration. Overall, our study underscores the potential for RDA and ROC curves to examine how providers interpret continuous diagnostic tests.

## Supporting information

Chockalingam et al. supplementary material Chockalingam et al. supplementary material

## Supplementary material

The supplementary material for this article can be found at https://doi.org/10.1017/ash.2025.72
