# Supplementary material for: Real-world interpretation of procalcitonin to guide antibiotic prescribing: a retrospective cohort study with regression discontinuity analysis
Source: Antimicrob Steward Healthc Epidemiol. 2025 May 28;5(1):e119. doi: 10.1017/ash.2025.72 (PMC12122395; doi:10.1017/ash.2025.72)

Supplementary Appendix

**Manuscript Title**: Real-World Interpretation of Procalcitonin to Guide Antibiotic Prescribing: A Retrospective Cohort Study with Regression Discontinuity Analysis

**Appendix content:**

Pg 2 ---- Supplement Table 1: Treatment decision categorizations
Pg 3 ---- Supplement Table 2: Procalcitonin values, stratified by antibiotic treatment decisions

Pg 4-5 --- Supplement Table 3: Supplemental Table 3: Sensitivity analysis for bandwidths around cut-points, including cut-point of 0.3

Pg 6 --- Supplement Figure 1a: University of Colorado Hospital antimicrobial stewardship procalcitonin algorithm

Pg 6 --- Supplemental Figure 1b: Comments included in procalcitonin result reporting
Pg 7 ---- Supplement Figure 2: Flow chart detailing the reasons for the encounter exclusions

| **Supplement Table 1: Treatment decision categorizations** |
| --- |
| **Full Antibiotic Course** |
| Receipt of antibiotics for at least four calendar days within any five-day period during hospitalization* |
| Prescription of a minimum four-day antibiotic course upon discharge |
| Continuation of the inpatient antibiotic regimen via a discharge prescription, totaling to at least four days of antibiotics |
| Number of days was unspecified on discharge prescription |
| Patient's death occurred while on antibiotics, even if course is fewer than four days |
| Treatment trimethoprim-sulfamethoxazole courses, determined by chart review |
| Single doses of Dalbavancin or Oritavancin |
| Course length of at least four days as determined by chart review for patients with end stage renal disease, given renally-adjusted dosing |
| **Partial Antibiotic Course** |
| Fewer than four days of antibiotics within any five-day period |
| Course length of fewer than four days as determined by chart review for patients with end stage renal disease, given renally-adjusted dosing |
| **No Antibiotics** |
| Single-agent azithromycin, rifaximin, dapsone, minocycline, and erythromycin courses as they are often used for non-infectious indications |
| Prophylactic trimethoprim-sulfamethoxazole courses, determined by chart review |
| Anti-tuberculosis drugs, otic and ophthalmic formulations, irrigation suspensions, topical antibiotics, antifungal agents, antiparasitic agents, antiviral agents, and nebulized antibiotics. |

| **Supplement Table 2: Procalcitonin values (ng/mL), stratified by antibiotic treatment decisions** | | | | |
| --- | --- | --- | --- | --- |
|  | Full Course of Antibiotics (n=3,018) | Incomplete Course / No Antibiotics (n=1,365) | Total (N=4,383) | *P*-value * |
| Mean (SD) | 3.3 (11.6) | 0.5 (3.6) | 2.4 (9.9) | <0.00001 |
| Median (IQR) | 0.29 (0.10, 1.24) | 0.08 (0.04, 0.19) | 0.18 (0.07, 0.72) |  |
| **Categorical procalcitonin lab result (ng/mL)** | | | | |
| < 0.1 | 737 (24.4%) | 774 (56.7%) | 1511 (34.5%) | <0.00001 |
| 0.1 - 0.24 | 672 (22.3%) | 348 (25.5%) | 1020 (23.3%) |  |
| 0.25 - 0.5 | 429 (14.2%) | 104 (7.6%) | 533 (12.2%) |  |
| > 0.5 | 1180 (39.1%) | 139 (10.2%) | 1319 (30.1%) |  |
| * *P*-values were calculated using chi-squared or Fisher's exact tests for categorical variables and unequal variances t-tests for continuous variables.  SD = standard deviation, IQR = interquartile range | | | | |

| **Supplemental Table 3: Sensitivity analysis for bandwidths around cut-points, including cut-point of 0.3** | | | |
| --- | --- | --- | --- |
| **Cut-point ng/mL ± bandwidth ng/mL; comparison ng/mL; (n)** | **Level of Care (LOC)** | **Odds Ratio (95% Confidence Intervals)** | **P Value** |
| 0.1 **±** 0.03; 0.08-0.10 vs 0.11-0.13; (677) | ICU | 0.73 (0.31 -1.74) | 0.48 |
|  | Stepdown | 0.63 (0.24 - 1.62) | 0.33 |
|  | Floor | 0.54 (0.26 -1.12) | 0.1 |
|  | All LOC | 0.60 (0.31 - 1.17) | 0.14 |
| 0.1 **±**0.05; 0.06-0.10 vs 0.11-0.15; (1161) | ICU | 0.63 (0.32 - 1.21) | 0.17 |
|  | Stepdown | 0.73 (0.36 - 1.50) | 0.39 |
|  | Floor | 0.70 (0.40 - 1.21) | 0.20 |
|  | All LOC | 0.69 (0.42 - 1.13) | 0.14 |
| 0.1 **±**0.1; 0.01-0.10 vs 0.11-0.20; (2354) | ICU | 0.76 (0.46 - 1.23) | 0.26 |
|  | Stepdown | 0.98 (0.58 - 1.66) | 0.94 |
|  | Floor | 0.92 (0.63 - 1.36) | 0.69 |
|  | All LOC | 0.89 (0.63 - 1.27) | 0.53 |
| 0.25 **±** 0.03; 0.22-0.24 vs 0.25-0.27; (220) | ***ICU*** | ***6.35 (1.06-38.06)*** | ***0.04*** |
|  | Stepdown | 1.96 (0.31 - 12.41) | 0.48 |
|  | Floor | 3.31 (0.79 - 13.95) | 0.10 |
|  | All LOC | 3.43 (0.93 - 12.64) | 0.06 |
| 0.25 **±** 0.05; 0.20-0.24 vs 0.25-0.29; (383) | ICU | 2.68 (0.75 - 9.50) | 0.13 |
|  | Stepdown | 1.47 (0.35 - 6.20) | 0.60 |
|  | Floor | 2.05 (0.73 - 5.77) | 0.17 |
|  | All LOC | 2.11 (0.83 – 5.40) | 0.12 |
| 0.25 **±** 0.1; 0.15-0.24 vs 0.25-0.34; (808) | ***ICU*** | ***3.56 (1.40 - 9.10)*** | ***0.01*** |
|  | Stepdown | 1.45 (0.56 - 3.76) | 0.44 |
|  | Floor | 2.0 (0.96 - 4.11) | 0.06 |
|  | ***All LOC*** | ***2.23 (1.16 – 4.27)*** | ***0.02*** |
| 0.3 **±** 0.03; 0.27-0.29 vs 0.30-0.32; (172) | ICU | 1.25 (0.15 - 10.61) | 0.84 |
|  | Stepdown | 0.67 (0.06 - 6.94) | 0.73 |
|  | Floor | 0.64 (0.11 - 3.69) | 0.62 |
|  | All LOC | 0.75 (0.15 – 3.86) | 0.74 |
| 0.3 **±** 0.05; 0.25-0.29 vs 0.30-0.34; (273) | ICU | 0.88 (0.15 - 5.14) | 0.89 |
|  | Stepdown | 0.32 (0.06 - 1.68) | 0.18 |
|  | Floor | 0.55 (0.15 - 2.05) | 0.37 |
|  | All LOC | 0.54 (0.16 – 1.81) | 0.32 |
| 0.3 **±** 0.1; 0.20-0.29 vs 0.30-0.39; (599) | ICU | 0.90 (0.28 - 2.86) | 0.86 |
|  | Stepdown | 0.45 (0.14 - 1.47) | 0.19 |
|  | Floor | 0.51 (0.22 - 1.19) | 0.12 |
|  | All LOC | 0.57 (0.26 - 1.23) | 0.15 |
| 0.5 **±** 0.03; 0.48-0.50 vs 0.51-0.53; (70) | ICU | 2.36E+07 (<0.001 - >1.8E+308) | 0.99 |
|  | Stepdown | 5.09E+07 (<0.001 - >1.8E+308) | 0.99 |
|  | Floor | 5.86 (0.19 -179.58) | 0.31 |
|  | All LOC | 8.82 (0.33 -233.86) | 0.19 |
| 0.5 **±** 0.05; 0.46-0.50 vs 0.51-0.55; (126) | ICU | 3.73E+07 (<0.001 - >1.8E+308) | 0.99 |
|  | Stepdown | 4.91E+07 (<0.001 - >1.8E+308) | 0.99 |
|  | Floor | 3.6 (0.29 - 43.20) | 0.32 |
|  | All LOC | 5.07 (0.49 - 52.08) | 0.17 |
| 0.5 **±** 0.1; 0.41-0.50 vs 0.51-0.60; (275) | ICU | 11.6 (0.92 -144.81) | 0.06 |
|  | Stepdown | 5.58 (0.65 - 47.50) | 0.12 |
|  | Floor | 2.80 (0.56 - 13.98) | 0.21 |
|  | All LOC | 3.57 (0.80 - 16.04) | 0.10 |

Supplement Figure 1a: University of Colorado Hospital antimicrobial stewardship procalcitonin algorithm


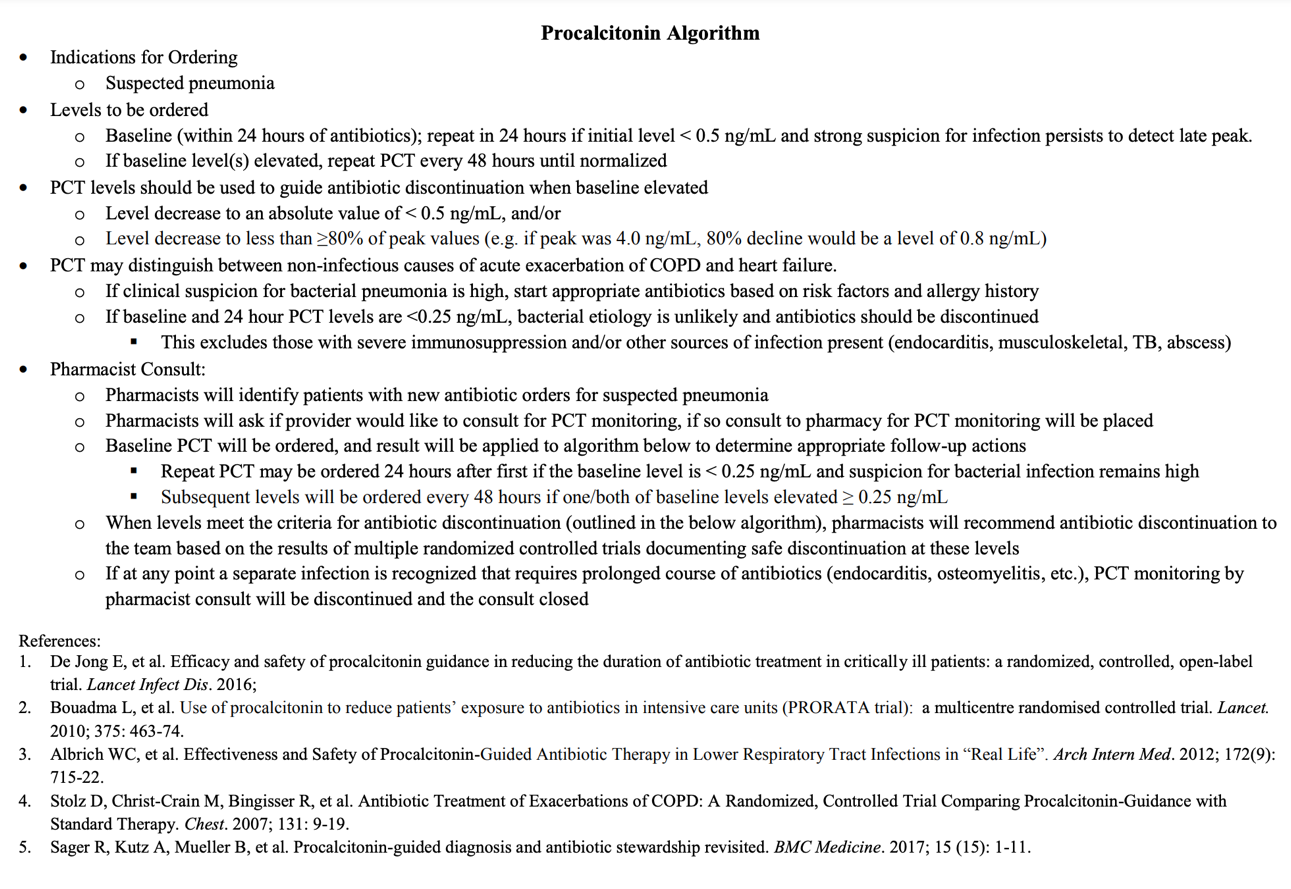


Supplement Figure 1b: Comments included in procalcitonin result reporting


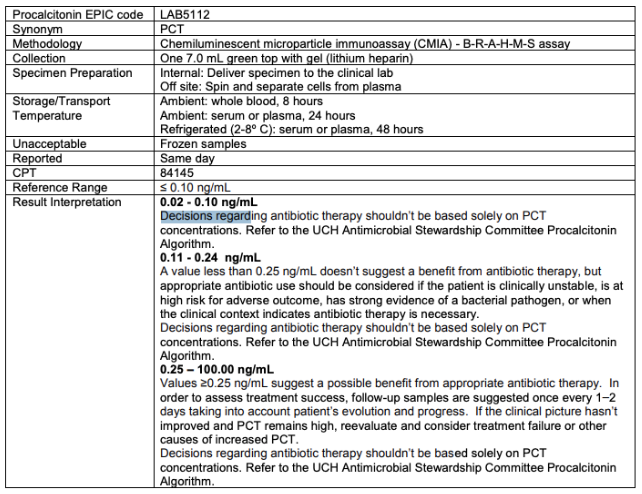


Supplemental Figure 2: Flow chart detailing the reasons for the encounter exclusions


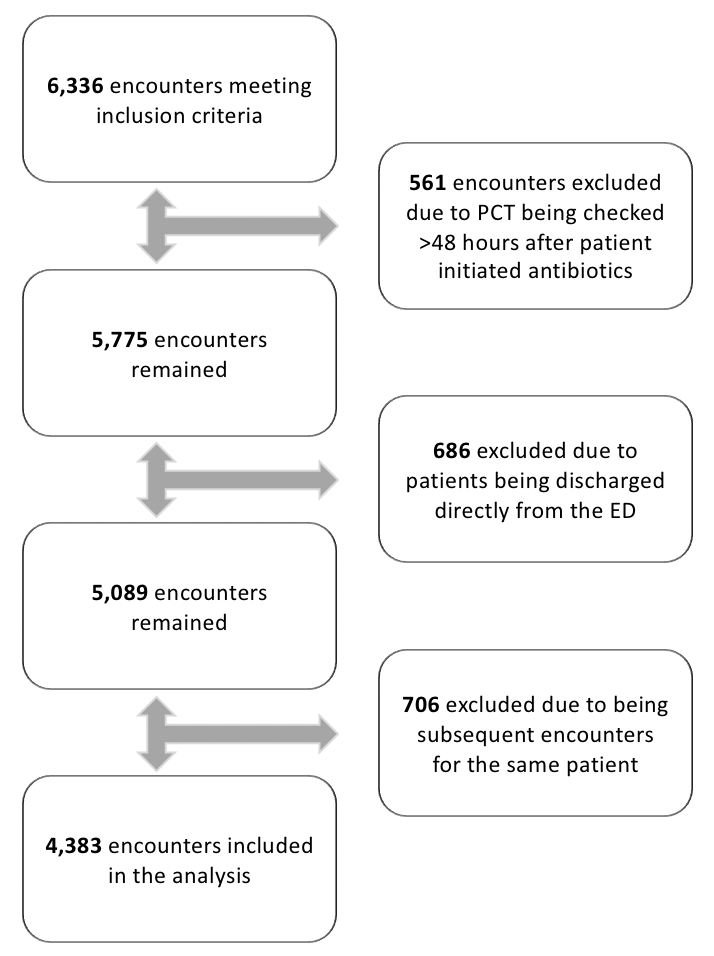

Supplement: Chockalingam et al. supplementary material [file S2732494X25000725sup001.docx]
